# Perspectives on Psoriasiform Adverse Events from Immune Checkpoint Inhibitors: Lessons Learned from Our Practice

**DOI:** 10.3390/medicina60030373

**Published:** 2024-02-22

**Authors:** Liliana Gabriela Popa, Calin Giurcaneanu, Mariana Georgiana Portelli, Mara Mădălina Mihai, Cristina Beiu, Olguța Anca Orzan, Ana Ion, Teodora Hrista Anghel

**Affiliations:** 1 Faculty of Medicine, ‘Carol Davila’ University of Medicine and Pharmacy, 020021 Bucharest, Romania; lilidiaconu@yahoo.com (L.G.P.); calin.giurcaneanu@umfcd.ro (C.G.); mariana-georgiana.ristea@rez.umfcd.ro (M.G.P.); mara.mihai@umfcd.ro (M.M.M.); cristina.beiu@umfcd.ro (C.B.); anaionoo@yahoo.com (A.I.); hrista-teodora.anghel@rez.umfcd.ro (T.H.A.); 2 Department of Dermatology, ‘Elias’ University Emergency Hospital, 011461 Bucharest, Romania

**Keywords:** psoriasis, psoriasiform adverse events, immune checkpoint inhibitors, immunotherapy

## Abstract

*Background*: New oncologic therapies, including immune checkpoint inhibitors (ICIs), have revolutionized the survival and prognosis of cancer patients. However, these therapies are often complicated by immune-related adverse effects (irAEs) that may impact quality of life and potentially limit their use. Among these adverse events are psoriasis and psoriatic arthritis that may develop de novo or flare under treatment with ICIs. Given the exceptional immune status of patients receiving ICIs, managing these conditions without interfering with the effect of the oncologic treatment may prove very challenging. Aim: To review the literature data on ICI-induced psoriasis exacerbation or development, to present our own experience, and to discuss the pathogenic mechanisms underlying this association and the optimal therapeutic approach for these patients. Case Reports: We report three cases of ICI-induced de novo psoriasis and two cases of ICI-induced psoriasis exacerbation that required systemic treatment. Oral acitretin treatment successfully controlled psoriasis lesions in three cases and allowed for the continuation of immunotherapy. *Literature Review*: We performed a medical literature search across several databases (PubMed, Medline, Google Scholar) using the search terms “immune checkpoint inhibitor-induced psoriasis/psoriasiform dermatitis/psoriasis arthritis”. We identified and revised 80 relevant publications that reported 1102 patients with psoriasis and/or psoriasis arthritis induced or exacerbated by ICIs. We assessed the type of cancer, the therapeutic agent involved, the clinical form of psoriasis, the presence or absence of psoriatic arthritis, the personal and family history of psoriasis, the age, the gender, the time until onset or exacerbation of skin lesions, the specific treatment recommended, the need for ICI discontinuation, and the patient’s outcome. *Conclusions*: As ICIs represent a fairly novel therapy, the association with several adverse effects is only now unraveling. Psoriasis exacerbation or onset following the initiation of immunotherapy is one such example, as more and more reports and case series are being published. Awareness of the relationship between psoriasis and treatment with ICIs, prompt recognition, and initiation of adequate skin-directed therapies are essential for the avoidance of skin lesions worsening, the need for systemic treatments that may interfere with ICIs’ effects, or the discontinuation of the latter. In the absence of generally accepted guidelines, it is advisable to treat patients with severe, widespread psoriasis with drugs that do not impair the effects of immunotherapy and thus do not alter the patient’s prognosis.

## 1. Introduction

The history of medical oncology has known several dramatic turning points, such as the discovery of X-rays in the late 1800s and that of cytotoxic drugs in the mid-1900s. Accumulating knowledge regarding the molecular characteristics of malignant tumors led to a new shift in the early 1980s, with the development of anticancer-targeted therapies. All these discoveries represented epochal advancements, greatly improving the survival of cancer patients. Still, a wide range of advanced or metastatic tumors lacked efficient treatment. Tremendous genetic engineering research brought about a new crucial moment and the beginning of a new oncologic treatment era, governed by immunotherapy. The concept of personalized, efficient, and well-tolerated anti-cancer treatment finally became a reality. The promising results of ongoing research focused on cell and gene therapy allow us to hope for even more efficient oncologic treatments [1].

The identification of molecules that downregulate the immune response, termed inhibitory immune checkpoints, has led to the development of a new class of oncologic drugs. Immune checkpoints prevent excessive and potentially detrimental immune reactions and promote self-tolerance, but are also stimulated by cancer cells, which thus avoid immune elimination [2].

The immune checkpoints ascertained so far are the adenosine A2A and A2B receptors, CD276, VTCN1, B and T lymphocyte attenuator (BTLA), cytotoxic T-lymphocyte-associated protein 4 (CTLA-4 or CD152), indole-amine 2,3-dioxygenase (IDO), killer-cell immunoglobulin-like receptor (KIR), lymphocyte activation gene-3 (LAG3), nicotinamide adenine dinucleotide phosphate oxidase isoform 2 (NOX2), programmed death 1 (PD-1) and its ligand PD-L1, T-cell immunoglobulin domain and mucin domain 3 (TIM-3), V-domain immunoglobulin suppressor of T cell activation (VISTA), sialic acid-binding immunoglobulin-type lectin 7 (SIGLEC 7 or CD328), and SIGLEC 9 (CD329) [3].

Immune checkpoint inhibitors (ICIs) are potent immune modulators able to restore anti-tumor immune response. These monoclonal antibodies have revolutionized cancer management as they have proven to be highly efficient in a variety of malignancies, succeeding in achieving durable responses while maintaining a favorable safety profile. The anti-CTLA-4 monoclonal antibody, ipilimumab, was the first ICI to be approved by the Food and Drug Administration (FDA) in March 2011 and by the European Medicines Agency (EMA) in November 2012 for the treatment of metastatic or unresectable melanoma. Since then, ten other ICIs have received approval from one or both drug regulatory authorities and their indications have greatly expanded. ICIs are classified into four categories, depending on their target: anti-PD-1 (nivolumab, pembrolizumab, cemiplimab, dostarlimab, retifanlimab), anti-PDL-1 (atezolizumab, avelumab, durvalumab), anti-CTLA-4 (tremelimumab), and anti-LAG-3 (relatlimab) monoclonal antibodies. They are used as monotherapy, in combination with other ICIs or molecular targeted therapy, as well as in association with chemotherapy or radiation therapy, and have remarkably improved the oncologic outcomes of patients with melanoma; Merkel cell carcinoma; cutaneous squamous cell carcinoma; basal cell carcinoma; head and neck cancer; lung, esophageal, gastric, colorectal, liver, kidney, bladder, breast, uterine, and cervical cancer; and sarcoma, lymphoma, and mesothelioma. Recently, they have been successfully used as neo-adjuvant therapy even in cancers hitherto not considered antigenic [4].

Nevertheless, given their unique mechanism of action, treatment with ICIs is often complicated by a particular spectrum of adverse events, most of which are immune-related (irAEs). More than 60% of ICI-treated patients experience one or more irAEs that can affect any organ. The resultant immune intolerance may manifest as cutaneous/mucous eruptions, thyroiditis, hypophysitis, hepatitis, pancreatitis, colitis, pneumonitis, myocarditis, uveitis, polyneuritis, etc. [5].

The most common irAEs are those involving the skin and mucous membranes. They occur in 18–34% of patients receiving PD-1/PD-L1 inhibitors and in 43–45% of patients treated with CTLA-4 inhibitors and encompass a wide range of disorders, from maculopapular or lichenoid rashes, pruritus/prurigo nodularis to autoimmune conditions (vitiligo, alopecia areata, bullous pemphigoid, and other immunobullous diseases, dermatomyositis, Sjogren syndrome), granulomatous disorders (pyoderma gangrenosum, sarcoidosis), eczematous or psoriasiform eruptions, erythema nodosum, Sweet’s syndrome, rosacea, and life-threatening conditions (Stevens–Johnson syndrome and toxic epidermal necrolysis) [6,7,8,9]. Fortunately, most cases are mild and readily manageable, with severe reactions having been reported in 1–3% of patients [5]. However, irAEs develop earlier and are more severe and persistent in patients receiving combined ICI treatment [10].

As ICIs represent a fairly novel therapy, the association with several adverse effects is only now unraveling. Psoriasis exacerbation or onset following the initiation of immunotherapy is one such example, as more and more reports and case series are being published. Psoriasis is a chronic, recurrent skin disease with a major impact on the patient’s quality of life. Given the exceptional immune status of patients receiving ICIs, managing severe psoriasis without interfering with the effect of the oncologic treatment may prove very problematic.

We review the literature data on ICI-induced psoriasis exacerbation or development, present our own experience (Table 1), and discuss the pathogenic mechanisms underlying this association and the optimal therapeutic approach for these patients.

## 2. Case Reports

### 2.1. Case 1

**Figure 1:**
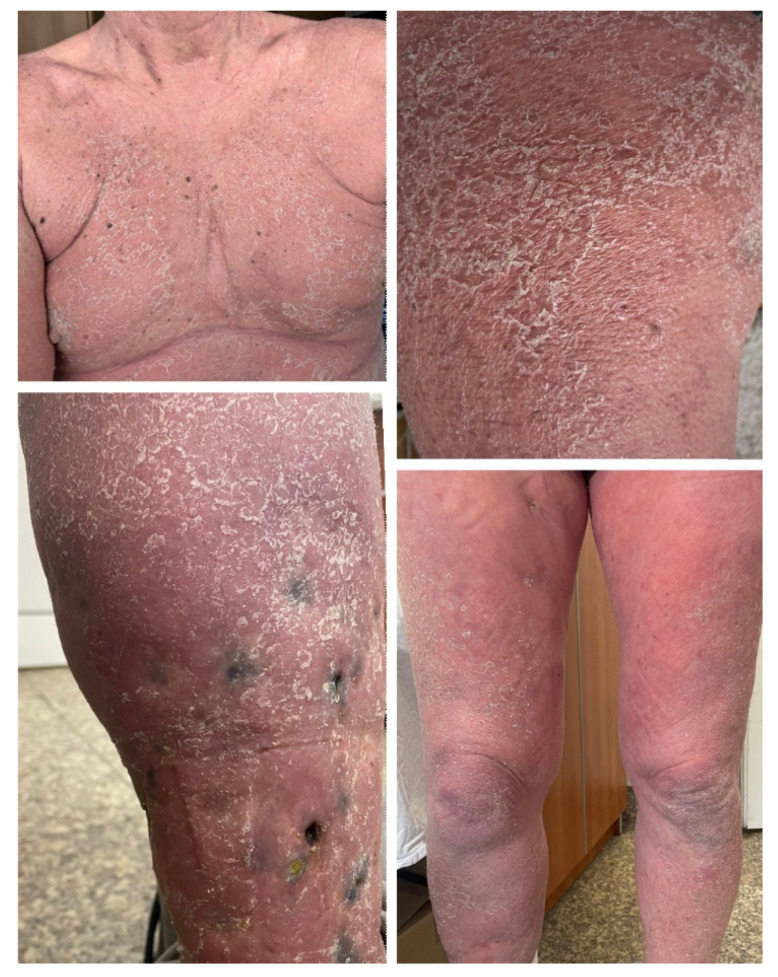
Case 1: Erythrodermic psoriasis—large, red patches covered with very thick silvery scales covering 90% of the body surface, and locoregional pigmented metastases (in transit) observed on the right shin in a patient receiving nivolumab and ipilimumab for advanced melanoma.

A 60-year-old male patient with no relevant family medical history and with a personal history of asthma and allergic rhinitis was diagnosed in March 2022 with locally metastatic regressive melanoma localized on the right lower limb. The primary tumor was surgically excised. In July 2022, a PET-CT scan revealed a metabolically active lesion in the right lung. The histopathologic examination of the lung tumor established the diagnosis of pulmonary tuberculosis, and in August 2022, the patient started tuberculostatic treatment. In September 2022, pembrolizumab was initiated, but numerous locoregional metastases soon developed. The patient underwent electrochemotherapy, with good results. In December 2022, pembrolizumab was replaced by nivolumab and ipilimumab combination therapy. Four days after the initiation of the combination therapy, erythematous and squamous plaques appeared on the trunk and limbs. The skin lesions generalized during the following 3 weeks. Topical corticoids and emollients, as well as systemic corticosteroids (methylprednisolone at an initial dose of 32 mg daily), were administered. He was referred to our clinic in February 2023 and was admitted for erythrodermic psoriasis (Figure 1). Treatment with acitretin 25 mg daily was initiated and systemic corticosteroid doses were tapered, along with intensification of the topical care that consisted of corticosteroids, keratolytic agents, and emollients. Combination therapy with ipilimumab and nivolumab was continued, with mild flares a few days after administration, and was followed by nivolumab monotherapy. The course of the skin lesions was favorable, with gradual clinical improvement and complete resolution with no other flares one month after cessation of the combined therapeutic regimen. Acitretin doses were gradually reduced and finally stopped without relapse of the skin lesions. Unfortunately, the patient succumbed due to progressive neoplastic disease.

### 2.2. Case 2

**Figure 2:**
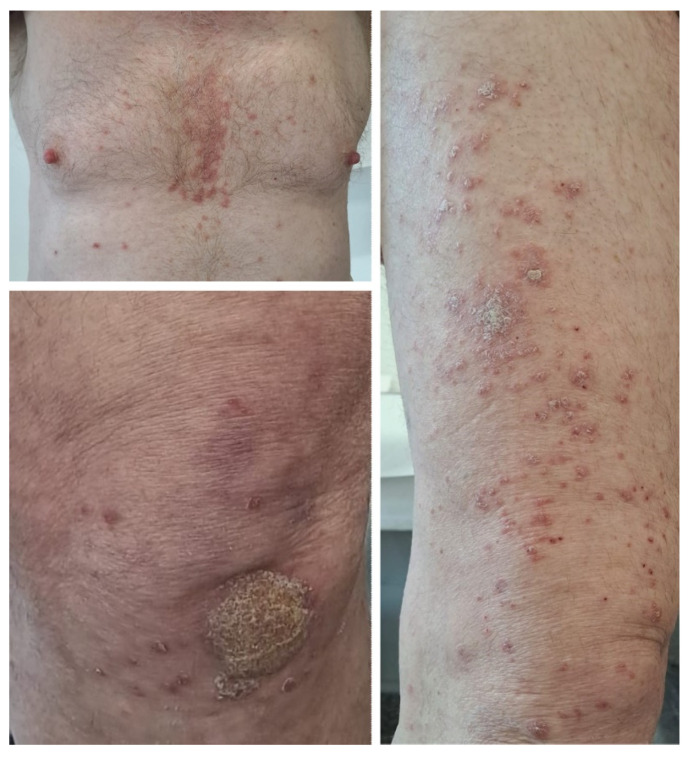
Case 2: Erythematous papules and plaques covered with micaceous silvery scales located on the trunk and extensor surfaces of the limbs in a patient who underwent treatment with avelumab for advanced urothelial cancer.

The second case is that of a 74-year-old male patient with no relevant family medical history and a personal history of chronic plaque psoriasis. The onset of psoriasis had taken place at the age of 30 and the disease had been stable and limited to the elbows and knees for the last several years. In April 2021, the patient was diagnosed with inoperable, locally advanced urothelial cancer. He underwent palliative chemotherapy with platinum salts and gemcitabine, followed by gemcitabine monotherapy, with stable partial remission of the neoplasm. In November 2022, immunotherapy with avelumab was initiated, and in December 2022, after the third dose of avelumab, the patient presented exacerbation of the skin lesions. He was referred to our clinic for widespread plaque psoriasis (Figure 2). Treatment with acitretin 20 mg daily and topical corticoids and keratolytic agents were recommended and the skin lesions slowly improved and have been stable during the past year, although the patient continued treatment with avelumab.

### 2.3. Case 3

**Figure 3:**
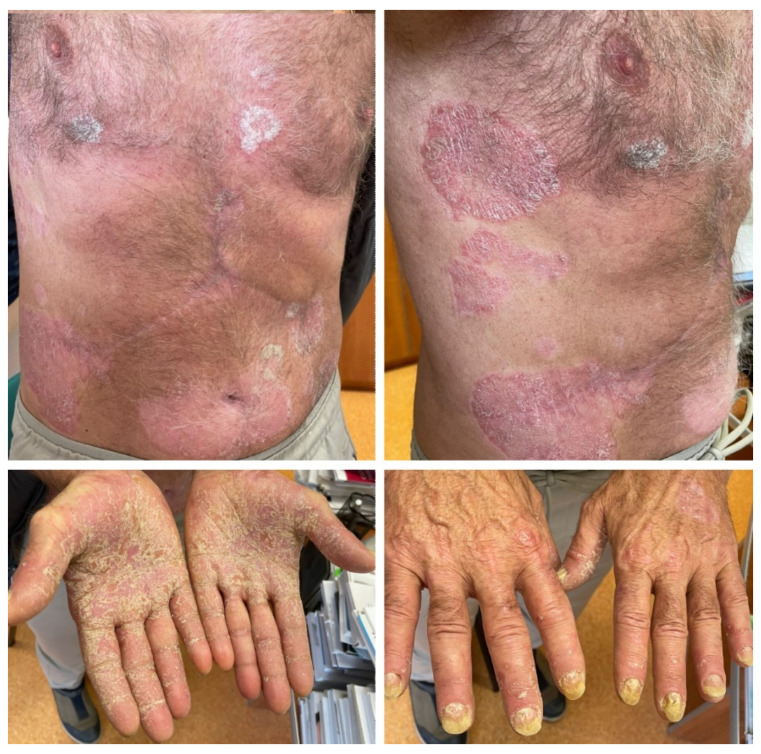
Case 3: Symmetrically distributed erythemato–squamous plaques located on the trunk and limbs, with significant palmar involvement and onychodystrophy in a patient who underwent treatment with atezolizumab and bevacizumab for advanced hepatocellular carcinoma.

The third case is that of a 66-year-old male patient with a 38-year history of psoriasis vulgaris and chronic hepatitis virus B and C infection, for which he had undergone pegylated interferon-α treatment and is currently receiving entecavir. The onset of psoriasis took place at the age of 28, after intense psychological stress. The patient presented generalized psoriasis skin lesions, which were controlled with topical corticoids keratolytic agents and phototherapy (psoralen and ultraviolet A radiation). After the initial episode, the skin disease had a very mild course, with the patient experiencing only two other flares in the context of viral infections that were easily managed with specific local treatment. No psoriasis skin lesions have appeared during the last 12 months. In January 2023, the patient was diagnosed with moderately differentiated hepatocellular carcinoma. An atypical liver resection was performed in February 2023, followed by the initiation of atezolizumab and bevacizumab treatment. Five months after the initiation of immunotherapy, he presented with generalized erythemato–squamous plaques, with severe palmar and plantar involvement and onychodystrophy (Figure 3). Treatment with acitretin 20 mg daily was recommended, associated with local corticosteroids, keratolytic agents, and emollients. Psoriasis skin lesions slowly improved while treatment with atezolizumab and bevacizumab was continued.

### 2.4. Case 4

**Figure 4:**
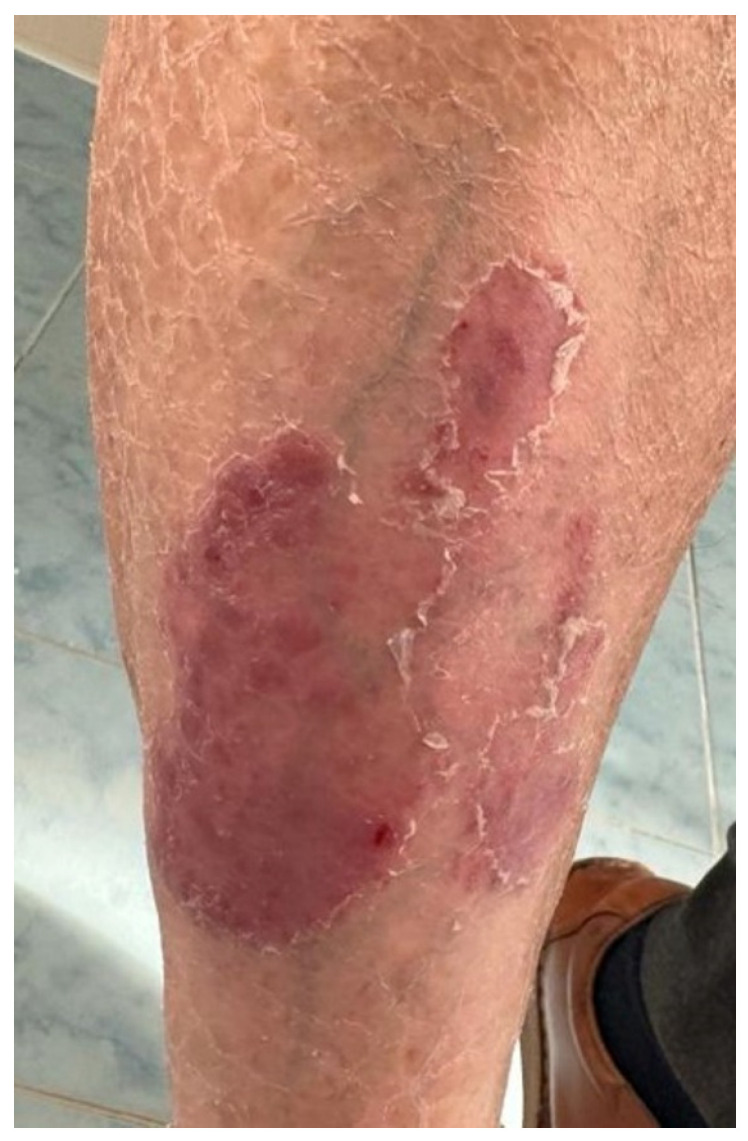
Case 4: Well-demarcated red scaly plaques on the shin of a patient with lung cancer that received treatment with pembrolizumab.

A 61-year-old male patient was referred to our clinic for well-demarcated erythemato–squamous plaques on the shins and abdominal area suggestive of chronic plaque psoriasis (Figure 4). The patient had no personal or family history of psoriasis. He was diagnosed in December 2020 with squamous lung cancer with metastases in both lungs and the left frontoparietal region. In January 2021, the patient started chemotherapy with paclitaxel, carboplatin, and immunotherapy with pembrolizumab, followed by pembrolizumab monotherapy. The cerebral metastasis was resected, and the patient also underwent radiotherapy. The patient responded well to chemotherapy and immunotherapy, with a stable disease. One year after the initiation of pembrolizumab, he developed the above-described skin lesions that were treated with topical corticosteroids, vitamin D analogs, and keratolytic agents. Immunotherapy was continued with rare flares of psoriasis lesions that only required local treatment.

### 2.5. Case 5

**Figure 5:**
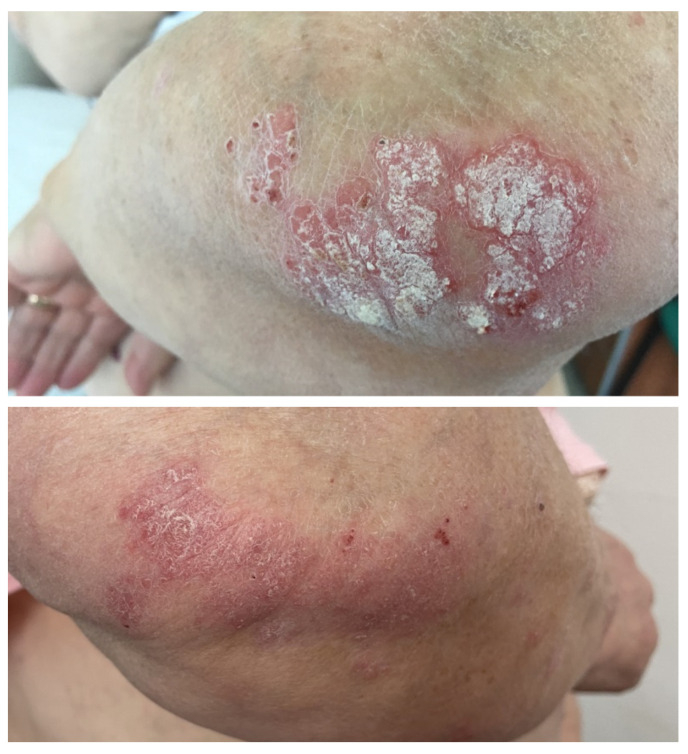
Case 5: Erythematous plaques with thick silvery scales on the surface distributed on the elbows before and after topical treatment in a patient treated with nivolumab for metastatic melanoma.

A 63-year-old female patient with no relevant medical history was diagnosed in July 2019 with cutaneous melanoma located in the left external malleolar region. An excisional biopsy was performed, and the histopathologic examination showed an ulcerated, superficial spreading melanoma with a vertical growth phase, and with a Breslow index of 5.2 mm and peritumoral lymphatic invasion—BRAF-negative. The patient underwent wide local re-excision, sentinel lymph node biopsy, and total body computed tomography scanning that revealed lymph node and pulmonary metastases. Immunotherapy with nivolumab was initiated soon after, to which the patient responded very well. Twelve months after the initiation of immunotherapy, the patient developed psoriasis vulgaris lesions located on the elbows and knees (Figure 5), which were controlled with topical corticoids and vitamin D analogs.

**Table 1:** Summary comprising the clinical findings of the case reports.

	Neoplasm	ICI	Age	Gender	Personal History of Psoriasis	Family History of Psoriasis	Time/Median Time until Psoriasis Onset	Type of Lesions	Psoriasis Treatment	Outcome
Case 1	Metastatic melanoma	Pembrolizumab first, then nivolumab + ipilimumab, then nivolumab monotherapy	60	Male	No	No	4 days after initiation of combination therapy	Erythrodermic psoriasis	Topical and systemic corticosteroids, acitretin 25 mg daily, keratolytic agents, emollients	Complete resolution of skin lesions, and treatment was continued. Patient died due to progressive metastatic disease
Case 2	Advanced urothelial cancer	Avelumab	74	Male	Yes, limited to elbows and knees	No	After third dose	Widespread plaque psoriasis	Acitretin 20 mg daily, topical corticoids, keratolytic agents	Lesions slowly improved, then stabilized, treatment was continued
Case 3	Hepatocellular carcinoma	Atezolizumab	66	Male	Yes, mild disease	No	5 months after initiation of immunotherapy	Generalized psoriasis with severe palmo–plantar involvment	Acitretin 20 mg daily, topical corticoids, keratolytic agents, emollients	Skin lesions showed slow improvement, treatment was continued
Case 4	Lung cancer	Pembrolizumab	61	Male	No	No	1 year after initiation of pembrolizumab	Plaque psoriasis	Topical corticoids, vitamin D analogs, keratolytic agents	Immunotherapy was continued, with rare flares of psoriasis lesions
Case 5	Metastatic melanoma	Nivolumab	63	Female	No	No	12 months after initiation of nivolumab	Plaque psoriasis on elbows and knees	Topical corticoids, vitamin D analogs	Immunotherapy was continued

## 3. Review of the Literature

According to PRISMA guidelines, we performed a medical literature search across several databases (PubMed, Medline, Google Scholar) using the search terms “immune checkpoint inhibitor-induced psoriasis/psoriasiform dermatitis/psoriasis arthritis”. We identified and revised 80 relevant publications and included the most important data provided by these studies in Table S1 [11,12,13,14,15,16,17,18,19,20,21,22,23,24,25,26,27,28,29,30,31,32,33,34,35,36,37,38,39,40,41,42,43,44,45,46,47,48,49,50,51,52,53,54,55,56,57,58,59,60,61,62,63,64,65,66,67,68,69,70,71,72,73,74,75,76,77,78,79,80,81,82,83,84,85,86,87,88,89,90]. We assessed the type of cancer, the therapeutic agent involved, the clinical form of psoriasis, the presence or absence of psoriatic arthritis, the personal and family history of psoriasis, the age, the gender, the time until onset or exacerbation of skin lesions, the specific treatment recommended, the need for ICI discontinuation, and the patient’s outcome.

A total of 1102 patients with psoriasis and/or psoriasis that was arthritis-induced or exacerbated by ICIs have been reported to date. Of these, 1068 patients presented psoriatic skin lesions. The exacerbation or de novo occurrence of psoriasis and/or psoriatic arthritis in ICI-treated patients was more common in males, with a male/female ratio of 2.76. The mean age of these patients was 66.81 years, with most of the cases belonging to the age group 61–70 years. Data regarding the family history of psoriasis were only available in twenty-five patients, with only three of the latter having a positive family history of psoriasis. As presented in Table S1, the onset or exacerbation of psoriasis and/or psoriatic arthritis complicated ICI treatment in patients with a wide variety of cancer types. It most frequently occurred in patients with lung cancer (227 cases, 27.8%), followed by patients with melanoma (82 cases, 10%), patients with urothelial cancer (22 cases, 2.7%), and, to a much lesser extent, in patients with hepatocellular carcinoma; head and neck squamous cell carcinoma; cavernous sinus squamous cell carcinoma; cutaneous squamous cell carcinoma; thyroid, breast, renal, digestive tract, uveal cancer, pharyngeal, pancreatic, ovarian, and bladder cancer; Merkel cell carcinoma; as well as Hodgkin’s lymphoma. Unfortunately, the type of cancer was not specified in 410 of the reported cases. The most common clinical form of psoriasis was plaque psoriasis, but ICI-induced palmoplantar, inverse, pustular, linear, rupioid, follicular, and nail psoriasis were also reported. Anti-PD1 agents more commonly induced psoriasis or psoriatic arthritis onset or flares compared to anti-PDL1 and anti-CTLA-4 agents. Among anti-PD1 agents, nivolumab was the culprit in most cases (one hundred and sixteen cases of psoriasis and seven cases of psoriatic arthritis), followed by pembrolizumab (fifty-nine cases of psoriasis and three cases of psoriatic arthritis) and cemiplimab (one case of psoriasis). Anti-PDL1 treatments were associated with psoriasis and/or psoriatic arthritis development or exacerbation in a much smaller number of cases, most of which occurred during treatment with atezolizumab (eighteen cases of psoriasis), followed by durvalumab (twelve cases of psoriasis) and avelumab (one case of psoriasis). Only one case of de novo psoriasis was reported in a patient undergoing treatment with ipilimumab. On the other hand, combination therapy with nivolumab and ipilimumab was complicated with psoriasis onset or flares in eight patients and psoriatic arthritis in three patients.

The psoriatic lesions imposed systemic treatment in 414 patients (37.5%). Of these, three hundred and fourteen patients (75.8%) received systemic corticosteroids, seventeen patients (4.1%) received methotrexate, and two patients (0.5%) were recommended cyclosporine for the control of skin lesions. Oral retinoids were used in 39 patients (9.4%). Novel therapies were employed in 42 patients (10.1%), the most frequently recommended being anti-PDE4 agents (apremilast), followed by anti-IL17, anti-TNFα, anti-IL23, and anti-IL12/IL23 agents.

Unfortunately, we only had information on the outcome of 154 cases because 63 patients discontinued ICIs, 53 of them due to uncontrolled psoriasis and/or psoriatic arthritis.

Table 2 serves as a comprehensive synthesis, encapsulating the key insights, findings, and data elucidated in the preceding section.

## 4. Discussion

Tumors represent an ample reservoir of antigens, both normal, overexpressed proteins and mutation-derived neoantigens, able to induce anti-tumor interferon (IFN) γ—mediated T-cell response, translated in a rich tumoral CD8^+^ T-cell infiltrate [91]. However, tumor cells evade the immune attack in various ways. One such way is the inhibition of cytotoxic T lymphocyte (CTL) function and proliferation by the expression of PD-L1 and a series of other receptors, which confer tumor cells anti-apoptotic properties. Moreover, in the setting of persistent high antigenic exposure, T cells upregulate inhibitory surface receptors, termed immune checkpoints, which prevent simultaneous signaling via different co-stimulatory molecules and exert a negative regulatory function meant to ensure self-tolerance. However, this attempt to maintain immunologic homeostasis also leads to tolerance towards certain tumor cells [92]. The most studied immune checkpoints are CTLA-4 and PD-1.

CTLA-4 is constitutively expressed by regulatory CD4^+^ T cells (Tregs) and inducibly expressed by CD4^+^ and CD8^+^ T cells upon activation [93]. It can also be found on the surface of some tumor cells. It is encoded by genes located in the proximity of the co-stimulatory receptor, CD28 locus on chromosome 2q33, and shares structural similarities with the latter, binding CD28′s ligands on dendritic cells (DCs) (CD80 and CD86) with greater affinity [94]. Thus, CTLA-4 inhibits T-cell proliferation and the release of IL-2 indirectly by blocking the stimulatory effect of CD28 binding. In addition, CTLA-4 binding to CD80 and CD86 leads to trans-endocytosis of its ligands and their removal from DCs’ surfaces [95].

PD-1 is found on the surface of activated CD4^+^ and CD8^+^ T cells, B cells, natural killer (NK) cells, monocytes, mast cells, DCs, and Langerhans cells. Its two ligands are differently distributed and upregulated, mainly by IFN-γ [96]. Hematopoietic cells (lymphocytes and myeloid cells), non-hematopoietic cells (endothelial and pancreatic cells), and some tumor cells express PD-L1, whereas PD-L2 is found on DCs, macrophages, mast cells, and peritoneal B1 cells [97]. Placental trophoblasts express both ligands, most probably implicated in fetal tolerance [98].

Initially, ICIs were considered to exert their effect merely by reactivation and proliferation of “predysfunctional” CTLs present in the tumor microenvironment (TME) upon inhibition of the interaction between CTLA4/PD-1 and their ligands [99,100]. This subset of CTLs, characterized by the expression of PD-1 and transcription factor TCF-1 may eventually differentiate into an irreversible “exhausted” non-functional phenotype [101,102].

This simplistic view has been revised and refined in the light of new findings. Several studies concluded that the response to the PD-L1 blockade primarily depends on PD-L1 expression by DCs, particularly DC1s, and to a lesser extent on its expression by tumor cells or infiltrating CTLs [103,104,105]. CTLA-4 also influences the early phase of T-cell priming, as its ligands are principally found on the DCs membrane [106].

The response to anti-PD-1 and anti-PD-L1 therapies revolves around the activity of DC1s present in the TME. IFN-γ released after the administration of anti-PD-1/anti-PD-L1 antibodies stimulates DC1s to produce interleukin (IL)-12b, which, in turn, promotes CD8^+^ T cell activation and tumor control [107].

Moreover, the effect of PD-1/PD-L1 inhibition is not limited to the TME but also impacts CD8^+^ T cell priming within tumor-draining lymph nodes (tdLNs) [108]. Migratory DCs present tumor antigens to naïve T cells in tdLNs, generating tumor-specific CTLs [109]. This initial phase, defined by independent activation of CD4^+^ and CD8^+^ T lymphocytes, is usually followed by a second step of priming. Cognate contact between CD4^+^ T helper (Th) cells and LN-resident DC1s enhances DC1s ability to induce proliferation and activation of CD8^+^ T cells [98,109,110]. It has been hypothesized that PD-1 binding during the first step of priming leads to the generation of helpless/predysfunctional CTLs that lack CD4^+^ T cell help [110]. They express inhibitory receptors (PD-1, CTLA-4, Tim-3, TIGIT, and LAG-3). The more such receptors on the surface of CTLs, the more dysfunctional these cells become [111]. Anti-PD-1 and anti-PD-L1 therapies temporarily restore the function of this cellular population and that of migratory DC1s both locally, within the tumor, and peripherally, in the tdLNs [98,112]. Nevertheless, they do not enable maturation into effector/memory CTLs as “reinvigorated” CTLs continue to express inhibitory receptors and ultimately convert to irreversible “exhausted” cells [101]. To complete the differentiation process, CTLs need costimulatory signals delivered by DCs through their surface receptors like CD28 and CD4^+^ T cell help signals, such as IL-12 [113,114].

ICIs induce T cell proliferation and increase their effector functions, their TCR diversity, and their reactivity against tumor antigens, as well as their resistance to Tregs [115,116]. Implicitly, the metabolic activity of T cells greatly intensifies, competing with tumor cells for nutrients [117]. Nevertheless, T-cell disinhibition is not only associated with a markedly enhanced anti-tumor response but is also responsible for the wide range of irAEs specific to ICI therapy. The rate, type, and severity of ICI-induced irAEs depend on the type of cancer, host factors, as well as the therapeutic agent since the immunologic impact of CTLA-4 and PD-1 blockade differs. ICIs may exacerbate previous inflammatory or autoimmune conditions. Animal studies showed that while CTLA-4 deficiency is associated with lethal, early-onset lymphoproliferative conditions [118], PD-1 deficiency generates more indolent autoimmune diseases [119,120].

Correspondingly, in clinical practice, irAEs are significantly more frequently encountered and more severe in patients treated with anti-CTLA-4 agents compared to patients undergoing anti-PD-1 treatment (27.3% vs. 16.3%). The risk of irAEs is greatest with combination therapy (55%), which is also associated with earlier, higher grade, and more persistent toxicities [121].

irAEs may affect any organ but dermatologic toxicities are the earliest (with a latency of 3–11 weeks) and most frequent side effects, occurring in over one-third of patients (34% of patients receiving PD-1 inhibitors and 43–45% of patients receiving CTLA-4 inhibitors) [5,92]. They are not dose-dependent and are similar to anti-CTLA-4 and anti-PD-1 antibodies, representing class adverse effects [92]. The most common cutaneous irAEs are maculopapular rashes, pruritus, and vitiligo [2]. Skin irAEs are generally mild and self-limiting, with severe cases not exceeding 2% [122].

Recently, reports and case series have drawn attention to psoriasis as a possible adverse effect of immunotherapy. Although most cases represent exacerbations of previously stable psoriasis, new-onset psoriasis lesions also occur during ICI treatment, generally 5–12 weeks after treatment initiation [40,68,123,124]. Any form of psoriasis may arise in ICI-treated patients, from chronic plaque psoriasis to guttate, pustular, palmoplantar, nail, scalp, inverse, and erythrodermic psoriasis [5]. These are clinically and histopathological indistinguishable from classic psoriasis. In addition, ICI-induced worsening or de novo psoriatic arthritis in the absence of a personal or family history of psoriasis has also been reported [81,125].

Psoriasis is the result of a complex interplay between genetic and environmental factors, between innate and adaptive immune responses, and a systemic inflammatory disease that echoes far beyond the skin. Th1 and Th17-cells play a central role in its pathogenesis. The mechanisms underlying ICI-induced psoriasis are incompletely understood. A series of hypotheses have been proposed.

The first hypothesis refers to the inhibitory effect that immune checkpoints exert on Th1/Th17 signaling [126] and the subsequent intense release of Th1 and Th17 cytokines (IL-2, IL-12, IFN-γ, and IL-17, IL-22, respectively) upon CTLA-4/PD-1 axis blockade [126]. This explains the exacerbation of preexistent subclinical inflammation in various organs, independent of the anti-tumor response.

Alternatively, the marked cytotoxic attack against tumor cells leads to the exposure of numerous host antigens and the potential generation of autoreactive CD4^+^/CD8^+^ T cells that target cross-reactive dermal/epidermal self-antigens, which have not yet been identified. This reaction is primarily mediated by IFN-γ [127].

Moreover, ICIs alter signaling between peripheral DCs and PD-1^+^ CD8^+^ T cells; therefore, antigen cross-presentation by DCs to CD4^+^T cells may take place [31].

Psoriatic keratinocytes have been shown to express low levels of PD-L1 and PD-L2 [126].

Another interesting finding in patients undergoing treatment with nivolumab is the elevated serum IL-6 level [89]. IL-6 may act as an autocrine regulator, promoting Th17 cell activation [31].

Pustular psoriasis exacerbated/induced by ICIs may follow the same pathogenic route as pustular psoriasis experienced by patients receiving TNF-α inhibitors. Loss of TNF-α-mediated control of autoreactive T cells or type 1 interferon production by plasmacytoid DCs has been proposed as a possible mechanism [127,128,129,130]. Viral infections may also represent precipitating factors, leading to the release of IFN-α from plasmacytoid DCs and the consequent stimulation of Th17 cells [131].

Many irAEs occur due to unleashed immune attacks against antigens, some of which are common for tumors and the affected tissue. Therefore, irAEs, especially cutaneous immune reactions, regardless of their severity, represent valuable markers predictive of tumor response to ICIs and a superior outcome [132]. Nonetheless, immunotherapy is often successful in patients who do not develop irAEs [133]. Numerous studies comparing overall survival (OS) and progression-free survival (PFS) in patients with or without irAEs have reported significantly improved rates in the former group of patients [134,135,136]. Furthermore, it seems that patients who experience irAEs in more than one system have an even better prognosis [137]. A recent meta-analysis confirmed the association between irAEs and improved survival regardless of the type of tumor, ICI, or irAE [138]. Still, these findings have been contradicted by the results of other studies and need further research [139]. ICI-induced or exacerbated psoriasis has also been correlated with treatment benefit, but further studies are needed to shed light on this association [84,89].

In most cases, irAEs are mild and easily manageable, but occasionally, patients develop severe, life-threatening immune adverse reactions to ICIs that require systemic immunosuppressive treatment and lead to dose reduction and even discontinuation of immunotherapy. The use of high doses of corticosteroids and immunosuppressive agents imposes much caution on these patients as they may interfere with the effect of immunotherapy and may cause tumor progression [138,140]. Interestingly, their impact on ICI efficacy depends on the moment during immunotherapy they are administered and the indication. The administration of immunosuppressants soon after the initiation of immunotherapy decreases its efficacy [140,141,142]. While corticotherapy prescribed for the alleviation of symptoms caused by the neoplastic disease proved to influence ICIs’ efficacy negatively, a series of studies concluded that systemic immunosuppressive therapy recommended for the control of irAEs does not influence the response rate to ICIs, OS, and PFS [125,141]. This, however, is still debatable.

As presented above, most cases of exacerbations or de novo psoriasis induced by ICIs are mild and respond to topical therapies, consisting of corticoids, calcineurin inhibitors, retinoids, vitamin D3 analogs, keratolytic agents, coal tar, anthralin, and emollients. Phototherapy is very efficient in controlling psoriasis flares but is a relative contraindication in melanoma patients.

Moderate and severe psoriasis pose a real therapeutic challenge as the majority of systemic treatments have immunosuppressive or immunomodulatory effects that may hinder the response to ICIs. On this account, we consider acitretin the drug of choice in such settings as it does not have immunosuppressive effects, and have successfully used it in our patients in doses of 20–30 mg daily. Moreover, acitretin exerts an intrinsic anti-tumor effect, being recommended in the prophylaxis of keratinocyte carcinomas and the treatment of lymphomas [142]. In selected cases, it may be used in combination with phototherapy, particularly narrow-band ultraviolet B (nbUVB), as it is less carcinogenic than UVA.

Methotrexate 10–25 mg weekly is also highly efficient for the treatment of psoriasis and psoriatic arthritis. The evidence available so far renders it safe in cancer patients, aside from a possible increased risk of non-melanoma skin cancer [142,143]. However, its influence on ICIs’ efficacy is not certain and needs to be studied further.

Moderate or severe psoriasis refractory to topical therapies occurring in ICI-treated patients has also been successfully treated with apremilast administered in an initial dose of 10 mg daily, which is gradually increased by 10 mg daily until day 5, followed by a maintenance dose of 30 mg BID [66]. Apremilast is not contra-indicated in cancer patients as it primarily affects innate immunity and scarcely influences the adaptive immune response. Although its impact on immunotherapy efficacy is not clear yet, it represents a valuable therapeutic alternative for cancer patients who develop psoriasis.

Systemic corticosteroids should only be recommended in short courses for the control of severe psoriasis flares, followed by slow tapering to avoid rebound.

Cyclosporine, another conventional psoriasis treatment, is not a choice in cancer patients due to its immunosuppressive, tumor-promoting effects.

As malignancy represents a contra-indication for biologic treatment, experience with this drug group in ICI-induced psoriasis is very limited. Anti-tumor necrosis factor (TNF) α agents are not recommended in these patients, not only because of their potent immunosuppressive effects and risk of cancer progression, but also due to a lack of efficacy that may be explained by a different pathogenic mechanism, not directly dependent on TNF α [92,129].

The safety of the newer classes of biologic agents used for the treatment of psoriasis, i.e., anti-IL 23, anti-IL 17, and anti-IL 12/23 monoclonal antibodies, in oncologic patients is also questionable. Both favorable outcomes [144] and loss of ICI anti-tumor efficacy [145] have been reported for both classes, but they should be used exclusively for severe, recalcitrant disease.

## 5. Conclusions

Along with the continuing advancement of novel oncologic therapies, a whole new spectrum of cutaneous and mucous side effects unfolds. Dermatologic adverse effects are the most common toxicities induced by immune checkpoint inhibitors and, though readily manageable in most cases, they can bring great discomfort, further alter the patient’s quality of life, and even be life-threatening either per se or by ICI discontinuation imposed by their severity. Awareness of the relationship between the myriad of dermatologic toxicities and treatment with ICIs, prompt recognition, and initiation of adequate skin-directed therapies are essential for the avoidance of skin and mucous membrane lesions worsening, the need for systemic treatments that may interfere with ICIs’ effects, or the discontinuation of the latter.

Psoriasis and psoriatic arthritis may develop or flare under treatment with ICIs as demonstrated by the numerous studies and case reports published so far. In the absence of generally accepted guidelines, it is advisable to treat patients with severe, widespread psoriasis with drugs that do not impair the effects of immunotherapy and thus do not alter the patient’s prognosis.

## Figures and Tables

**Table 2:** Comprehensive summary of reviewed information.

Number of patients	Total number of patients—1102 Patients with psoriatic skin lesions—1068 Patients with psoriatic arthritis—31 Patients with unspecified psoriasis/psoriatic arthritis—29		
Gender distribution	Male—509 Female—184 Unspecified—409 Male/female ratio—2.76		
Age distribution	Mean age—66.81 Most affected age group—61–70		
Types of neoplastic disease	Lung cancer (227 cases, 27.8%) Melanoma (82 cases, 10%) Urothelial cancer (22 cases, 2.7%) Head and neck scuamocellular carcinoma (19 cases, 2.3%) Renal cancer (19 cases, 2.3%) Hepatocellular carcinoma (11 cases, 1.3%) Digestive tract cancer (5 cases, 0.6%) Other specified cancer (15 cases, 1.8%) Unspecified neoplasia (419 cases, 51.2%)		
Most common ICI-induced type of psoriasis	Plaque psoriasis		
Type of ICI inducing psoriasis/psoriatic arthritis		Psoriasis	Psoriatic arthritis
	Nivolumab	116	7
	Pembrolizumab	59	3
	Cemiplimab	1	0
	Unspecified anti-PD-1	151	1
	Atezolizumab	18	0
	Avelumab	1	0
	Durvalumab	12	0
	Unspecified anti-PD-L1	20	0
	Ipilimumab	1	0
	Ipilimumab + Nivolumab	8	3
	Unspecified ICI	402	18
Treatment needed for psoriatic lesions	Systemic treatment (37.5%), of which: Systemic corticosteroids 75.8%. Novel therapies 10.1%. Oral retinoids 9.4%. Methotrexate 4.1%. Cyclosporine 0.5%.		Topical treatment (62.5%)

## Data Availability

Data are contained within the article.

## Supplementary Materials

The following supporting information can be downloaded at: https://www.mdpi.com/article/10.3390/medicina60030373/s1, Table S1: Summary comprising the clinical findings of the case reports.
