# Supplementary material for: Perspectives on Psoriasiform Adverse Events from Immune Checkpoint Inhibitors: Lessons Learned from Our Practice
Source: Medicina (Kaunas). 2024 Feb 22;60(3):373. doi: 10.3390/medicina60030373 (PMC10972058; doi:10.3390/medicina60030373)
Supplement: Supplementary file 1 [file medicina-60-00373-s001.zip › medicina-2869400-supplementary.pdf]

Table S1 – Summary of the important findings considered in the review

Nr.	Study / report	Neoplasm	ICI	Psoriasis patients	Age / mean age	Gender	Personal history of psoriasis	Family history of psoriasis	Time / median time till psoriasis onset	Psoriasis treatment	Outcome
1.	Kochi Y et al., May 2023 [1]	lung cancer	pembrolizumab	1 case of simultaneous development of generalized pustular psoriasis and pemphigoid	70	M	NO	N/A	9 months after cessation of pembrolizumab due to a complete response of the lung tumor	anti-IL17 antibodies and oral corticosteroids 10mg/day	remission of both skin diseases
2.	Belzer A et al., May 2023 [2]	N/A	N/A	13 psoriasiform eruptions in psoriasis cohort<br>19 psoriasiform eruptions in control cohort	68	10 F<br>8 M<br>In psoriasis grou cohort	YES in 18 patients	N/A	N/A	N/A	N/A
3.	Hansen I et al., May 2023 [3]	stage IIB metastatic melanoma	nivolumab	1 patient with plaque psoriasis	67	M	NO	NO	after the second treatment	anti-TNFα antibodies (infliximab)	complete remission after 5 doses<br>ICI discontinued after the 6th infusion at patient's request (at which point

											infliximab was initiated)
4.	L'Orphelin JM et al, Apr 2023 [4]	unresectable stage IV melanoma	anti PD-1 or combination therapy (anti PD-1 and anti-CTLA4 or anti-lag)	19 psoriasiform rashes out of 63 patients with inflammatory benign dermatoses	N/A	73.3 % M	N/A	N/A	N/A	topical and oral corticosteroids retinoids (3 patients), apremilast (1 patient)	N/A
5.	Gargiulo L et al., Apr 2023 [5]	metastatic cutaneous melanoma	pembrolizumab	1 case of severe plaque psoriasis	28	M	YES	N/A	1 months after the start of treatment (2 doses)	Anti-IL23 antibodies (risankizumab)	complete remission; pembrolizumab was continued with no psoriasis flares
6.	Hussain K et al., Mar 2023 [6]	3 cutaneous melanomas<br>1 NSCLC<br>1 pharyngeal SCC<br>1 cavernous sinus SCC<br>1 lung adenocarcinoma	4 pembrolizumab,<br>1 durvalumab,<br>1 cemiplimab,<br>1 ipilimumab+ nivolumab, followed by maintenance nivolumab	7 patients with chronic plaque psoriasis and 1 with rupi-oid psoriasis	70.2 years	4F, 3M	YES in 5 patients	N/A	median time to psoriasis exacerbation was 5.2 weeks; median time to de novo psoriasis onset was 26 weeks		ICI treatment was permanently discontinued in two cases, held in two cases and then resumed, and continued in two patients; one patient declined further treatment.
		MM	ipilimumab+ nivolumab, followed by maintenance nivolumab		65	M	NO	N/A		corticosteroids, apremilast	immunotherapy held and then restarted

		NSCLC	pembrolizumab		71	F	YES	N/A		oral prednisolone	back to baseline CPP; immunotherapy held and then restarted
		NSCLC	durvalumab		75	M	YES	N/A		oral prednisolone followed by acitretin	treatment discontinued due to disease progression
		MM	pembrolizumab		61	F	YES	N/A		topical treatments	back to baseline CPP
		MM	pembrolizumab		73	F	YES	N/A		oral prednisolone	pembrolizumab discontinued due to progression of disease, not skin-related symptoms
		SCC of the left hypopharynx	pembrolizumab		72	M	NO	N/A		UVB phototherapy	excellent response after 27 sessions; declined further treatment
		SCC of the cavernous sinus	cemiplimab		75	F	YES	N/A		topical treatments	treatment continued
7.	Gleason L et al., Feb 2023 [7]	metastatic hepatocellular carcinoma	atezolizumab	1 case of psoriasiform eruption	63	M	YES	N/A	a week following the third cycle atezolizumab + bevacizumab	anti-IL17 antibodies (ixekizumab)	after initiating ixekizumab, atezolizumab was restarted without a flare of the cutaneous eruption; Complete remission of the psoriasiform

											eruption in 4 months
8.	Dorman K et al., Feb 2023 [8]	metastatic alveolar soft part sarcoma	pembrolizumab and axitinib	stable chronic psoriasis	80	M	YES	N/A	N/A	N/A	ICI was continued
9.	Nikolaou VA et al., Dec 2022 European Academy of Dermatology and Venereology Task Force of Dermatology for Cancer Patients [9]	111 NSCLC<br>25 melanoma<br>8 head&neck SCC<br>7 renal cancer<br>11 urothelial cancer<br>1 Hodgkin lymphoma<br>1 Merkel cell carcinoma<br>5 hepatocellular cancer	148 anti-PD-1 agents<br>20 anti-PD-L1 agents	175 patients (23% of 762 ICI treated patients)	66.5	116 M, 59 F	N/A	N/A	8.75 doses	N/A	N/A
10.	Lim JH et al., Nov 2022 [10]	hepatocellular carcinoma	atezolizumab	1 case of psoriasis	68	M	NO	N/A	1-2 weeks	topical treatment	initial amelioration, with recurrence 3 weeks after the second treatment;

											atezolizumab was discontinued and the lesions disappeared gradually
11.	Jfri A et al., Sep 2022 [11]	lung cancer (4) thyroid cancer breast cancer urothelial cancer (2) melanoma (2) cutaneous SCC esophageal cancer tongue SCC		262 patients with ICI-induced psoriasiform eruption (3% of 8863 ICI treated patients) 13 patients with inverse psoriasis (5%)	72	7/13 (53 %) male	YES in 4 patients<br>NO in 9 patients		3.5 weeks till flare 7 weeks till development		8 complete remissions, 4 partial responses with infusion-related flares;treatment was held in one patient for one cycle.
		breast cancer	pembrolizumab		77	F	NO	N/A	6 weeks	topical treatment	
		lung cancer	atezolizumab		65	F	YES	N/A	4 weeks	topical treatment	
		thyroid cancer	nivolumab+ipilimumab		72	M	NO	N/A	4 weeks	topical treatment	
		lung cancer	nivolumab		78	F	NO	N/A	2 weeks	topical treatment	
		urothelial cancer	pembrolizumab	psoriasiform eruption	69	M	NO	N/A	4 weeks	acitretin	
		melanoma	ipilimumab	psoriasiform eruption	37	M	NO	N/A	3 weeks	topical treatment	
		lung	nivolumab+i	chronic	65	F	YES	N/A	4 weeks	topical	

		cancer	pilimumab	plaque psoriasis						treatment	
		urothelial cancer	atezolizumab	psoriasiform eruption	81	M	NO	N/A	5 weeks	topical treatment	
		melanoma	nivolumab+ipilimumab	psoriasiform eruption	51	F	NO	N/A	3 weeks	apremilast	
		cutaneous SCC	nivolumab	psoriasiform eruption	77	M	NO	N/A	4 weeks	topical treatment	
		esophageal cancer	pembrolizumab	scalp psoriasis	73	F	YES	N/A	NA	topical treatment	
		tongue SCC	pembrolizumab	chronic plaque psoriasis	67	M	YES	N/A	6 weeks	methotrexate	
		lung cancer	pembrolizumab	psoriasiform eruption	81	M	NO	N/A	3 weeks	topical treatment	
12.	Kase M et al, Sep 2022 [12]	1 metastatic renal carcinoma	nivolumab + ipilimumab	psoriasis + arthritis	55	M	YES	N/A	3 cycles of combined Nivolumab + ipiliumumab	discontinuation of ICI<br>Oral prednisolone, apremilast, topical combination of active vitamin D3 and glucocorticoids	discontinuation of ICI
		1 metastatic renal carcinoma	pembrolizumab	psoriasis	50s	M	YES	N/A	3 weeks afer introducing pembrolizumab	apremilast	psoriatic lesions relieved
		1 metastatic non-small cell lung	durvalumab	psoriasis	69	M	NO	N/A	after second cycle of durvalumab	topical combination of vitamin D3 analog and	durvalumab discontinued after 5 cycles<br>lesions subsided in 2

		carcinoma								corticosteroid	months
13.	MA VT et al., Jun 2022 [13]	metastatic melanoma	1 patient on nivolumab + ipilimumab, then nivolumab monotherapy	plaque psoriasis + arthritis	59	M	NO	N/A	after 4 cycles of nivolumab monotherapy	prednisone, hydroxychloroquine- minimal improvement, then anti-IL17 antibodies (secukinumab)	gradual improvement of his arthralgias; antineoplastic therapy stopped
		metastatic melanoma	1 patient on pembrolizumab	plaque psoriasis + arthritis	75	M	YES	N/A	N/A	anti-IL17 antibodies (secukinumab)	complete resolution of his psoriatic lesions and dramatic improvement in arthralgias; Pembrolizumab continued
14.	Mohta A et al., May 2022 [14]	locally advanced urothelial carcinoma	atezolizumab	1 case of rupioid psoriasis	40	M	NO	N/A	after 2 months (2 cycles of therapy)	oral acitretin	good response; atezolizumab temporary discontinued
15.	Seervai RNH et al., May 2022 [15]	metastatic esophageal adenocarcinoma	nivolumab	1 case of severe pustular psoriasiform eruption	58	F	NO	N/A	3 days after initiation of nivolumab and progressing to confluent erythroderma with pustules over 2 weeks	refractory to topical and systemic steroids, acitretin Response to ustekinumab	discontinuation of nivolumab

16.	Wong PY et al., Mar 2022 [16]	advanced NSCLC	atezolizumab (single dose) plus chemotherapy	1 case of psoriasis that did not flare	N/A	N/A	YES	N/A	N/A	N/A	remarkable response of the neoplasm without developing psoriasis flares
17.	Tang K et al., Feb 2022 TriNetX Diamond Network [17]	neoplasms of digestive organs, bronchus or lung, melanoma of skin, and urinary tract	anti-PD1 anti-PDL1	7008 eligible patients who developed cirAEs after treatment with anti-PD-1 or anti-PD-L1 therapy, from which 299 psoriasis patients	68.3	3961 M (56.5%) 3044 F (43.3%) 10 unknown (0.14%)	N/A	N/A	N/A	N/A	N/A
18.	Tirpack A et al., Dec 2021 [18]	metastatic pancreatic cancer	pembrolizumab + leuprolie	psoriasis + PsAflare	62	M	YES	N/A	one year after starting pembrolizumab	methotrexate + presnisone	very good skin and joint response
19.	Çelik U et al., Dec 2021 [19]	lung carcinoma melanoma colon carcinoma	3 patients on nivolumab<br><br>1 patient on durvalumab	4 patients with psoriasis	57.7 +/- 10.5	N/A	N/A	N/A	N/A	topical treatment + phototherapy	N/A
20.	Onishi Y et al., Nov 2021 [20]	NSCLC	atezolizumab	palmoplantar pustulosis	75	M	NO	NO	10 months	topical treatment	poor response, after 2 months atezolizumab was discontinued because the patient developed fever, and his skin symptoms

											subsequently improved. Two months later, his irAE had resolved. Atezolizumab was reintroduced, and then his skin symptoms worsened
21.	Furuta H et al., Nov 2021 [21]	advanced lung adenocarcinoma	nivolumab	1 case of palmoplantar pustulosis	75	M	NO	N/A	after 28 cycles of nivolumab	Systemic costicosteroids	Nivolumab treatment was temporary stopped and resumed after improvement of skin lesions. However, his palmoplantar pustulosis worsened until disease progression with nivolumab therapy. Atezolizumab was administered as the third-line treatment, and palmoplantar pustulosis worsened, accompanied by an eruption on the trunk. With atezolizumab showing marginal efficacy, nab-paclitaxel treatment was started as the fourth-line agent.

											Skin lesions completely disappeared with discontinuation of ICI use
22.	Halle BR et al., Oct 2021 retrospective cohort study, patients from eight academic centers [22]	62 patients (82%) with melanoma<br>5 patients (7%) with lung cancer<br>2 patients (3%) with head and neck cancer<br>7 patients (9%) with other cancers	51 (67%) received anti-PD-1 antibodies<br>8 (11%) anti-CTLA-4<br>17 (22%) combination treatmentanti-PD-1/CTLA-4	76 patients with pre-existing psoriasis, most frequently plaque psoriasis (46 patients (61%) and 15 (20%) with psoriatic arthritis.<br>43 patients (57%) experienced a psoriasis flare of cutaneous (39 patients) and/or extracutaneous disease (arthritis and iritis- 7 patients). 3 had both cutaneous	67	50 (66%) M<br><br>26 F	YES	N/A	44 days	Of those who experienced a flare:<br>23 patients (53%) were managed with topical therapy<br>16 (21%) needed systemic therapy<br><br>Of the 35 patients with **cutaneous involvement only**:<br>20 had partial/complete remissions under topical therapies ±phototherapy<br>3 received acitretin	Only 5 patients (7%) required immunotherapy discontinuation for psoriasis flare – all with melanoma<br><br>Forty-five patients (59%) experienced other irAEs, 17 (22%) of which were grade ¾<br>Other irAEs resulted in ICI discontinuation in 22 patients<br><br>Overall, 20 patients (26%) were rechallenged with ICIs or received additional treatment with ICIs after their initial treatment regimen

				and extracutaneous flares						2 received prednisone monotherapy<br>2 received acitretin + prednisone<br>1 received apremilast + prednisone<br>1 received apremilast monotherapy<br>3 patients with cutaneous flare resolved without treatment.<br><br>All 3 patients with **concurrent cutaneous and extracutaneous flares** were treated with topical agents<br><br>The 2 patients with **grade 3 arthritis** exacerbation required additional systemic	

										treatment:<br>1 prednisone<br>1 prednisone + methotrexate<br><br>4 patients with an isolated flare of **PsA, all grade 2**, of which 3 improved with prednisone and 1 with NSAIDs	
23.	D'Erme AM et al., Sep 2021 [23]	advanced melanoma	pembrolizumab	1 case of plaque psoriasis	72	F	NO	NO	after 1 month	Topical and systemic corticosteroids + acitretin	good response, patient was able to continue immunotherapy
24.	Calvo V et al.,Jul 2021 [24]	lung cancer	Nivolumab (2)<br>nivolumab+ carboplatin/ paclitaxel (1)	3 patients with preexisting psoriasis, 1 with exacerbation of psoriasis during treatment	N/A	N/A	YES,	N/A	N/A	corticosteroids	1 patient discontinued treatment due to worsening of psoriasis<br>1 patient discontinued due to disease progression<br>1 patient continued treatment
		N/A	Nivolumab (1)<br>nivolumab+ipilimumab (1)	3 de novo psoriasis	N/A	N/A	NO	N/A	N/A	N/A	N/A

			nivolumab+ carboplatin/ paclitaxel (1)								
25.	Killion L et al., Aug 2021 [25]	1 patient with esophageal carcinoma	nivolumab	plaque psoriasis	60	M	NO	NO	after 4 cycles widespread	acitretin	partial response
		1 patient with renal cell carcinoma	nivolumab	plaque psoriasis	73	M	NO	NO	N/A	acitretin	PASI 90 in 6 weeks
26.	Jatwani K et al., Aug 2021 [26]	advanced SCLC	nivolumab	Psoriasis flare and PsA	65	F	YES, stable scalp psoriasis	N/A	12 weeks after initiation of nivolumab after 24 weeks: psoriasiform rash on the scalp and retroauricular area	oral prednisone	improvement of symptoms, nivolumab was continued
27.	Mullangi S et al., Jun 2021 [27]	metastatic renal cell carcinoma	nivolumab	1 case of palmoplantar psoriasis	66	M	NO	N/A	after 7 months	apremilast and retinoids	nivolumab was continued through these symptoms, but three months later, the patientdeveloped severe diarrhea, requiring systemic steroids and infliximab. Nivolumab was held

											at the time. He continued to stay off therapy without recurrence of disease at his last follow-up two years after stopping nivolumab
28.	Brown LJ et al., May 2021 retrospective study of patients with advanced melanoma and pre-existing autoimmune disease in 10 international centers from March 2015 to February 2020 [28]	advanced melanoma	46 patients on ipilimumab + nivolumab 9 patients on ipilimumab + pembrolizumab	3 of 6 patients with preexisting psoriasis experienced a flare	63 for the whole study group	26 (47 %) M 29 (53 %) F	YES	N/A	19 days (4-167) for the whole study group	systemic corticosteroids ±immunosuppressive agents	N/A
29.	Thompson LL et al., May 2021 [29]	N/A	anti-PD1 anti-PD-L1 anti-CTLA4	Of 358 patients who developed cirAE, 12 patients with psoriasis	N/A	N/A	N/A	N/A	N/A	N/A	N/A
30.	Nikolaou V et al., May 2021 Retrospective study by	NSCLC 69 (60%) melanoma 17	**anti-PD1: 99 (86.1%)** nivolumab 68 (59.2%)	115 patients grade 1, 2, and 3 disease severity was	M - 68.5	M - 88 (76.5%)	YES in 33 (30.8 %)	data available in 93	mean number (SD) of drug dosages until psoriasis onset	only topical agents in 69 patients (60%) nb	29 patients (25.9%) interrupted and 20 (18%) permanently discontinued ICIs

	ENCADO, 9 institutions [30]	(14.8%) head & neck SCC 6 (5.2%) renal cell carcinoma 6 (5.2%) urothelial carcinoma 6 (5.2%) Hodgkin's Lymphoma 2 (1.7%) Merkel cell carcinoma 1 (0.9%) hepatocellular carcinoma 3 (2.6%) gastric cancer 2 (1.7%) mesothelioma 1 (0.9%) ovarian cancer 1 (0.9%) pulmonary	pembrolizumab 30 (26.1%) spartalizumab 1 (0.8%)<br><br>**anti-PDL1: 16 (13.9%)** durvalumab 6 (5.2%) atezolizumab 9 (7.9%) avelumab 1 (0.8%)	reported in 60 of 105 (57.1%, 10 missing data), 34 of 105 (32.4%), and 11 of 105 (10.5%), respectively. The ratio between exacerbation and de novo cases was 1:4.3.<br><br>Plaque psoriasis was the most commonly diagnosed clinical form (49/115, 42.6%), followed by the palmoplantar (14/115, 12.2%)<br><br>Interestingly, 30/115 (26.1%)	F- 63. 7	F - 27 (23. 5%)	cases, of which 20 presebted active disease at ICI initiation	patients, 32 of which had family history of psoriasis	or exacerbation was 11.2 (14.9). In patients with clinically present psoriasis upon ICI initiation, deterioration of the disease was recorded sooner compared to those with no active psoriatic at baseline (mean number of infusions 5.4 vs 12.2, p<0.05)	UVB+topical steroids in 4 (3.5%) systemic therapy required in 47 patients (40.9%): acitretin (21 patients, 18.3%) systemic steroids (8 patients, 7%) apremilast (7 patients, 6.1%) methotrexate (5 patients, 4.3%) biologics (4 patients, 3.6%): infliximab (1), adalimumab (1), ustekinumab (1), guselkumab (1)	because of psoriasis.<br><br>Guttate psoriasis and grade 2 or 3 disease were significant positive predictors for antitumor response of ICI, whereas pruritus was a negative predictor.<br><br>18 out of 23 patients treated with acitretin showed either excellent response of psoriasis with complete clearance of the lesions (6/23, 26%) or partial response (12/23, 52.1%).<br><br>Excellent / partial response or partial were reported in all patients treated with apremilast.<br><br>All patients treated with systemic steroids showed a

		neuroendocrine cancer 1 (0.9%)		patients developed simultaneously or subsequently more than one clinical subtypes of psoriasis.<br><br>Nail involvement was recorded in 37/115 cases (32.7%)<br><br>8.1% reported symptoms of PsA.							positive clinical response<br><br>Partial response was observed in one patient in infliximab and in one patient in ustekinumab, whilst two individuals did not response to adalimumab and guselkumab.<br><br>The median time (CI) to response to psoriasis treatment was<br>8 months for acitretin<br>14 months for apremilast<br>6 months for methotrexate<br>3 months for steroids.
31.	Gonzalez-Mazón I et al., May 2021 [31]	of 102 patients treated with ICI, 13 developed cutaneous manifestation	N/A	1 patient with de novo psoriasis<br>2 patients with flares of preexisting psoriasis<br>1 patient with flare of	60.2 for the whole study gro	N/A	YES in 2 cases of psoriasis and 1 case of	N/A	N/A	topical treatment for the patient who developed de nvo psoriasis during ICI treatment	complete remission

				preexisting PsA	up		PsA				
32.	Mayor Ibarguren A et al., Mar 2021 [32]	advanced uveal melanoma	nivolumab	3 patients with plaque psoriasis	50	M	NO	NO	after 5 months	apremilast	good response; nivolumab continued to date
		advanced laryngeal carcinoma		2 patients with severe inverse prosiasis	70	M	YES	N/A	after 2 weeks	apremilast	good clinical response; tumor progression occurred after 10 months of nivolumab and apremilast treatment, so both were discontinued
		advanced squamous lung cell carcinoma		1 patient with psoriasis and PsA	60	M	YES	N/A	after 1 week	apremilast	positive clinical response; After 10 months of follow-up, tumor progression occurred
33.	Glinos GD et al., Mar 2021 [33]	stage III melanoma	nivolumab	1 patient with generalized plaque psoriasis	61	F	NO	NO	onset 2 weeks after the first infusion, worsening after the second infusion, evolving into generalized erythematous plaques with overlying scale involving 80% body surface	antiI-L23 antibodies (risankizumab)	rapid and durable response of skin lesions; nivolumab was discontinued after 2 cycles

34.	Cutroneo P S et al., Mar 2021 Eudravigilance during the period between the date of market licensing (for each study drug) and 30 October 2020 [34]	N/A	ipilimumab + tremelimumab nivolumab (175; 55.9%) pembrolizumab (104; 33.2%) atezolizumab(15; 4.8%), durvalumab avelumab (1; 0.3%) cemiplimab (2; 0.6%) ipilimumab (12; 3.8%) nivolumab + ipilimumab (7; 2.22%)	315 cases of psoriasis or psoriasiform reactions (3.8% of 8213 reports of cutaneous ADRs)	66.3% in the age range 65-85	75.9 % M	YES in 70.8% caases	N/A	?<br>N/A	145 patients (46.3%) were treated with topical agents<br>24 (7.7%) were treated with phototherapy<br>18 (5.7%) requiredsystemic steroids<br>10 (0.3%) required specific antipsoriatic systemic treatments:<br>2 acitretin<br>1 cyclosporine<br>1 methotrexate<br>1 efalizumab<br>2 etanercept,<br>3 apremilast. Of these, 7 were treated with nivolumab and 3 with pembrolizumab.	unknown outcome in 124 cases (39.3%)<br>60 cases (19%) “recovered/ resolved” cases were generally considered<br>78 cases (24.7%) “recovering/resolving”<br>2 cases (0.6%) were resolved “with sequelae”<br>51 cases (16%) were “not recovered/not resolved
35.	Foti C et al., Jan 2021 [35]	Metastatic melanoma	nivolumab	1 case of severe psoriasis	62	M	YES	N/A	3 weeks after the first course of nivolumab	apremilast	slight improvement in 3 months, PASI 90 at 12 weeks; after 42

				exacerbation					treatment		months of therapy with nivolumab and apremilast, diseases were stable.
36.	Umeda Y et al., Nov 2020 [36]	NSCLC	pembrolizumab	1 case of plaque and pustular psoriasis	68	M	YES, stable	N/A	53 days after initiating anti-PD-1 therapy, with progressive aggravation till 90% BSA affected	cyclosporin A + systemic prednisolone	discontinuation of anti-PD-1 therapy; substantial improvement of psoriatic lesions, flares of pustular psoriasis in the following months
37.	Siciliano MA et al., Oct 2020 [37]	metastatic melanoma	pembrolizumab	1 case of severe plaque psoriasis	75	M	NO	N/A	after 10 cycles	apremilast + methylprednisolone, progressively reduced until discontinuation	Pembrolizumab was interrupted for 4 weeks until the improvement of skin lesions and the disappearance of itching<br>After 10 months, the patient had a good general clinical condition, complete response of the tumor and psoriasis complete remission.
38.	Marti-Marti I et al., Sep 2020 [38]	metastatic lung squamous cell carcinoma	pembrolizumab	1 case of rupioid psoriasis	67	M	YES, stable	N/A	2 months after initiating the treatment with pembrolizumab	acitretin	good response; anti-PD1 therapy temporary discontinued, pembrolizumab was not reintroduced due to evidence of

											disease progression
39.	Lin WH et al., Sep 2020 [39]	lung squamous cell carcinoma	durvalumab	annular psoriasiform eruption	73	M	NO	NO	after 7 months of treatment with durvalumab	nb-UVB + topical treatment	durvalumab was temporarily ceased for two doses, then resumed amelioration of psoriasis lesions in 2 months, no recurrence of psoriasis lesions after resuming durvalumab
40.	Corneli P et al., Sep 2020 [40]	metastatic NSCLC	atezolizumab	1 case of inverse psoriasis	76	M	NO	NO	1 week after starting atezolizumab	cessation of atezolizumab	complete resolution of axillary lesions; atezolizumab was discontinued due to tumor progression
41.	Huang PW et al., Aug 2020 [41]	lung suamous cell carcinoma	pembrolizumab	linear psoriasis	71	M	NO	NO	2 weeks after the first infusion	topical treatment	good response; pembrolizumab was discontinued due tot umor progression
42.	Nigro O et al., Jun 2020 [42]	metastatic epithelioid cell melanoma	Ipilimumab + nivolumab for 5 months, then nivolumab monotherapy	1 case of psoriasis and PsA	39	M	YES for psoriasis	N/A	after 26 cycles	apremilast	clinically significant response in 3 months remained suspended; nivolumab was stopped due to the appearance of disabling acute polyarthritis
43.	Mao M et al., May 2020 [43]	metastatic NSCLC<br><br>Stage IV	atezolizumab	1 case of severe psoriasis, with plaques	53	M	YES, STABLE	NO	2 weeks after atezolizumab + nab-paclitaxel initiation	topical treatment	Response of psoriasis lesions in 2 months; atezolizumab was

		Adenocarcinoma		and guttate lesions							discontinued
44.	Di Altobrando A et al., May 2020 [44]	metastatic melanoma	nivolumab	1 case of severe new-onset palmoplantar and nail psoriasis	59	M	NO	NO	during the first 4 months	topical treatment	psoriatic cutaneous lesions completely disappeared and marked nail improvement was observed; nivolumab was temporary ceased, then restarted
45.	Takama H et al., Apr 2020 [45]	bladder cancer	pembroliumab	Psoriasis vulgaris	74	M	NO	N/A	2 weeks after the third cycle	apremilast	skin lesions resolved in 2 months, then apremilast was stopped with no recurrence; pembroliumab was discontinued after third cycle because of immune-mediated interstitial pneumonia
46.	Suzuki M et al., Mar 2020 [46]	stage IV lung adenocarcinoma	pembrolizumab	psoriasiform dermatitis	78	F	NO	N/A	after 3 cycles	oral prednisolone	psoriasiform dermatitis gradually improved; the patient refused pembrolizumab readministration due to concerns about worsening of psoriasiform dermatitis
47	Guven D et al., Jan 2020	SCLC	nivolumab	1 case of de novo	51	F	NO	NO	1 week after the fourth cycle	topical treatment	psoriasis plaques regressed;

	[47]			psoriasis							immunotherapy was continued without complications
48.	Politi A et al., Jan 2020 [48]	advanced squamous lung carcinoma	nivolumab	1 case of psoriasis flare	71	F	YES	N/A	after the first cycle	acitretin + PUVA	after the third cycle, the extensive skin plaques required cessation of immunotherapy; amelioration of skin lesons afterwards
49.	Tison A et al., Dec 2019- retrospective cohort study from January 2017 to January 2018 via 3 French national networks of experts in oncology and autoimmunity. Adults with preexisting autoimmune disease who were receiving ICIs were assessed	melanoma (20 patients) NSCLC (10 patients) urologic cancer (1 patient)	ipilumab (5 patients nivolumab (19 patients) pembrolizumab (5 patients) ipilimumab+ nivolumab (2 patients)	The study included 112 patients<br><br>31 patients with preexisting psoriasis or PsA, of which 25 had flares	65	N/A	YES in 31 patients, of whom 17 had active disease at start of immunotherapy	N/A	N/A	7 patients (33%) required systemic treatment: glucocorticoids (3 patients) methotrexate (3 patients) acitretin (3 patients)	permanent discontinuation of ICI due to immunotoxicity in 6 patients<br><br>death in 7 patients, none due to irAEs<br><br>doar pso sau toti?- doar pso (tabelul 3 din celalalt word)

	for the occurrence of flare of preexisting autoimmune disease, other IRAEs, and cancer response. [49]										
50.	Hara T et al., Dec 2019 [50]	NSCLC	pembrolizumab	1 case of psoriasis flare and de novo PsA	69	M	YES, stable plaque psoriasis	N/A	after the third cycle	oral prednisolone + salazosulfapyridine	significant clinical improvement after 6 weeks; pembrolizumab was discontinued
51.	Monsour EP et al., Oct 2019 [51]	NSCLC	pembrolizumab	1 case of psoriasis flare	63	F	YES	N/A	3 months after Initiation of pembrolizumab	Anti-IL17 antibodies (secukinumab)	immunotherapy was continued successfully with the co-administration of secukinumab without complications or the recurrence of NSCLC
52.	Nikolaou V et al., Dec 2019 [52]	Lung cancer (84) Breast cancer (129) Colorectal cancer (59)	Immune checkpoint inhibitors = 48 (10.5%)	459=total number of patients, of which: 10 psoriasis patients 1 patient with	60.6 years mean age for the	M 28 (15.6%) F 20 (7.7%)	YES in 2 male patients	YES in 5 male patients	N/A	N/A	N/A

		Kidney cancer (16) Melanoma (44) Ovarian cancer (19)		psoriasis + PsA	whole study group						
53.	Scarfi F et al., Aug 2019 [53]	advanced NSCLC	pembrolizumab	1 case of follicular psoriasis exacerbation	69	M	YES, psoriasis in remission for several years	N/A	after the 4th infusion	oral prednisolone	rapid improvement and complete remission in 1 month; pembrolizumab was continued
54.	Johnson D et al., Jun 2019 [54]	advanced melanoma (stage IIIC)	pembrolizumab	1 case of psoriasiform eruption	80	M	NO	NO	12 weeks after the initial dose of pembrolizumab	antiI-L17 antibodies (secukinumab)	marked improvement in 4 weeks, but secukinumab was discontinued after 3 doses due to thrombocytopenia Pembrolizumab was discontinued. A new metastasis occurred, but was stable 28 weeks after secukinumab treatment, without re-initiation of pembrolizumab
55.	Santos-Juanes J et al., Jun	advanced urothelial	atezolizumab	1 case of plaque	75	M	YES, stable	N/A	12 days after the first infusion of	oral prednisone +	skin lesions resolved in 3 weeks and no

	2019 [55]	carcinoma		psoriasis flare					atezolizumab	bilastine	residual lesions were observed; atezolizumab was discontinued because of the skin toxicity
56.	Fattore D et al., Mar 2019 [56]	non-metastatic NSCLC	nivolumab	1 case of psoriasis exacerbation	74	F	YES, psoriasis in remission for several years	N/A	6 months after ICI initiation	apremilast	remarkable clinical improvement of skin lesions in 6 weeks, without any obvious interference with immunotherapy
57.	Rios A et al., Jan 2019 [57]	hepatocellular carcinoma	nivolumab	1 case of grade IV psoriasiform dermatitis	62	M	N/A	N/A	after 10 doses of nivolumab	etoposide	dramatic response after only 7 days: nivolumab was discontinued and a new oncologic treatment was started
58.	De Bock M et al., Aug 2018 [58]	metastatic melanoma	nivolumab	1 case of psoriasis flare	65	F	YES, scalp psoriasis	N/A	after 11 nivolumab infusions	topical treatment	successful control of the psoriatic lesions; nivolumab interval was extended from 2 to 3 weeks
59.	Troyanova-Slavkova S et al., Aug 2018 [59]	metastatic base of the tongue squamous cell carcinoma	nivolumab	1 case of psoriasis	58	M	NO	NO	4 weeks after initiation of nivolumab	nbUVB + acitretin	complete remission of skin lesions; nivolumab was continued
60.	Cortellini A et al., Mar 2018 [60]	metastatic melanoma	sequential ipilimumab, pembrolizu	1 case of chronic plaque	62	M	YES	N/A	N/A	topical treatment	ICI was continued with no significant toxicities

			mab, and nivolumab	psoriasis that did not exacerbate							
61.	Lidar M et al., Mar 2018 [61]	advanced melanoma (12 patients) endometrial carcinoma (1 patient) sinunasal carcinoma (1 patient)	nivolumab (4 patients) ipilimumab (1 patient) pembrolizumab (8 patients) nivolumab + ipilimumab (1 patient)	PsA<br><br>psoriasis (1 patient)	61 ± 11 s	F-57%	YES in 1 patient	YES in 1 patient	average time to onset of a rheumatic iAE in our series was 11.2 months	systemic steroid therapy methotrexate	N/A
62.	Danlos FX et al., Mar 2018 retrospective study of the REISAMIC registry of grade ≥2 irAEs occurring in ICI-treated patients [62]	melanoma (36 patients) NSCLC (6 patients) other cancers (3 patients)	pembrolizumab (34 patients) nivolumamb (10 patients) avelumab (1 patient)	12 patients with preexisting cutaneous psoriasis (including one with PsA), of which 4 patients with psoriasis flares	63.3 (pt tot lotul)	M - 46.7 %<br><br>(pt tot lotul)	YES in 12 patients	N/A	2.1 months between ICI initiation and the irAE (pt tot lotul)	Immunosupresive drgus 10/20 patients with irAE	Complete resolution of the irAE 2.8/20 patients
63.	Chujo S et al., Mar 2018 [63]	NSCLC	nivolumab	1 case of psoriasis	75	M	NO	NO	after the 6th infusion of nivolumab	topical treatment	psoriatic eruptions significantly improved; nivolumab was continued
64.	Voudouri D et al., Nov 2017	NSCLC (3	pembrolizumab (1	5 cases of psoriasis:	65.8	Male –	YES for 2	YES for 2	after 1-6 cycles	nb-UVB + topical therapy	4 patients continued immunotherapy and

	[64]	patients) papillary urothelial carcinoma (1 patient) tonsil squamous cell carcinoma (1 patient)	patient) nivolumab (2 patients) durvalumab (2 patients)	1 patient with active psoriatic lesions at the time of treatment initiation 4 patients withd guttate psoriasis the most severe exacerbation being associated with durvalumab treatment		80%	patien ts	patie nts		in 4 patients methotrexate + prednisolone in 1 patient with psoriasis and PsA	1 patient discontinued durvalumab was forced to
65.	Sugiura Y et al., Sep 2017 [65]	advanced NSCLC	nivolumab	1 case of de novo psoriasis and PsA	66	M	NO	N/A	after 4 courses of nivolumab	oral predni-solone	nivolumab was continued, but the lung cancer progressed
66.	Elosua-González M et al., Aug 2017 [66]	metastatic NSCLC	nivolumab	1 case of palmoplantar psoriasis and PsA	68	M	NO	NO	after the 3rd nivolumab infusion	methotrexate + tapering dose of oral prednisone	skin lesions and joint symptoms gradually resolved over 9 months of therpy; nivolumab was not restarted (progression of lung cancer on CT)
67.	Ruiz-Bañobre J et al., Jun	metastatic NSCLC	nivolumab	1 case of PsA and plaque	47	M	YES, stable	NO	arthralgias after 8 infusions;	NSAIDs + low dose	Amelioration in 9 weeks; nivolumab

	2017 [67]			psoriasis flare			psoriasis		diagnosis of PsA after 11 cycles of nivolumab	methylprednisolone + methotrexate	was continued and antitumor therapy efficacy was not influenced
68.	Okiyama N et al., 2017 [68]	metastatic melanoma	nivolumab	2 cases of de novo psoriasiform dermatitis	78.5	M	NO	N/A	after 11 cycles of nivolumab	oral retinoid in 1 case<br>oral prednisolone in 1 case	Near complete remission of skin lesions; nivolumab was discontinued
69.	Ruiz-Bañobre J et al., Mar 2017 [69]	metastatic renal cell carcinoma	nivolumab	1 case of de novo psoriasis	45	M	NO	NO	2 weeks after the first course of nivolumab; worsened after the 3rd infusion	topical treatment	Good clinical response; nivolumab was stopped for 21 days and reinitiated when the rash improved to grade 1
70.	Nonomura Y et al., Feb 2017 [70]	melanoma	nivolumab	3 cases of de novo or exacerbated psoriasis vulgaris	N/A	N/A	N/A	N/A	N/A	N/A	N/A
71.	Menzies AM et al., Feb 2017 Retrospective study of patients with advanced melanoma and preexisting autoimmune diseases and major prior irAEs to	advanced melanoma	anti-PD-1	4 of 8 patients experienced a flare (3 out of 6 patients with psoriasis, 1 out of 2 patients with PsA)	71 for the whole group	M 31 (60 %)	YES	N/A	38 days	oral steroids, MTX, apremilast	10 (20%) continued ICIs<br>8 (15%) temporary discontinued ICIs<br>2 (4%) permanently discontinued ICIs<br><br>pentru tot lotul

	ipilimumab from 13 academic tertiary referral Centers [71]										
72.	Murata S et al., Jan 2017 [72]	metastatic amelanotic malignant melanoma	nivolumab	1 case of de novo psoriasis	89	M	NO	N/A	2 weeks after the first course of nivolumab, worsened after the second course	topical treatment	well controlled
73.	Schmutz JL, Dec 2016 [73]	NSCLC	nivolumab	1 case of de novo psoriasis and PsA	80	M	NO	N/A	after the 8th perfusion	methotrexate + prednisone	improvement of psoriasis and PsA in 1 month, progressive decrease of doses (MTX & Prednisone); nivolumab was discontinued for 1 month, then reintroduced with a continued response and without paoriasis recurrence
74.	Law-Ping-Man S et al., Nov 2016 [74]	stage IV NSCLC	nivolumab	1 case of de novo psoriasis and PsA	80	M	NO	NO	after the 8th nivolumab infusion	oral methotrexate + low dose oral prednisone	after 1 month of therapy, both psoriatic skin lesions and joint symptoms gradually resolved, allowing gradual tapering of

											methotrexate and prednisone; nivolumab was discontinued for 4 weeks and restarted with continued response and without recurrence of psoriasis or PsA
75.	Kato Y et al., Oct 2016 [75]	oral mucosal metastatic melanoma	nivolumab	1 case of plaque psoriasis flare	65	M	YES, no treatment in the last months	N/A	3 weeks after the first course of nivolumab	oral etretinate	Skin lesions improved immediately; nivolumab was continued
76.	Chia PL et al., Jun 2016 [76]	metastatic NSCLC	pembrolizumab	1 case of psoriasis flare	74	M	YES	N/A	after 2 cycles (6 weeks)	topical treatment + phototherapy	slow improvement over the next few months: immunotherapy was ceased
77.	Totonchy MB et al., May 2016 [77]	metastatic melanoma	pembrolizumab	inverse psoriasiform eruption	80	F	NO	N/A	between cycles 2 and 3 of pembrolizumab	topical treatment	rapid clinical improvement; pembrolizumab eas resumed
78.	Sahuquillo-Torralba A et al., Apr 2016 [78]	metastatic NSCLC	pembrolizumab	1 case of psoriasis flare	67	M	YES, stable	N/A	after the 1st cycle - progressive worsening and erythrodermic psoriasis after	acitretin	Significant improvement of skin lesions after 45 days; pembrolizumab was continued, with no other flares,

									the 2nd cycle		maintaining a PASI 4 for 3 months after the initial outbreak
79.	Matsumura et al, Feb 2016 [79]	metastatic melanoma	nivolumab	1 case of psoriasis flare	87	M	YES	N/A	after the 2nd infusion of nivolumab	oral prednisolone	psoriatic lesions gradually improved, and prednisolone was gradually tapered without recurrence of psoriasis; nivolumab was discontinued
80	Ohtsuka M et al, Jul 2015 [80]	oral mucosal melanoma	nivolumab	1 case of de novo psoriasiform eruption	80	M	NO	NO	after the 4th dose of nivolumab	oral prednisolone	Rapid improvement of skin lesions, which recurred upon prednisolone tapering; continued 0.1 mg/kg prednisolone; nivolumab was discontinued
